# Investigation of the Microstructure and Mechanical Performance of GH4099 Alloy Fabricated by Selective Laser Melting

**DOI:** 10.3390/ma18102271

**Published:** 2025-05-14

**Authors:** Bo Chen, Yilong Zhong, Wenying Li, Yanying Li, Qiyou Wang, Yingjie Lu, Zichen Qi, Shenqi Wang, Yanbiao Li

**Affiliations:** 1 College of Mechanical Engineering, Zhejiang University of Technology, Hangzhou 310023, China; chenb@zjut.edu.cn (B.C.); 17770124010@163.com (Y.Z.); qiyou.wang.zjut@gmail.com (Q.W.); 15868846514@163.com (Y.L.); qizichen@zjut.edu.cn (Z.Q.); wangshenqi1102@163.com (S.W.); 2 State Key Laboratory of Fluid Power and Mechatronic Systems, Zhejiang University, Hangzhou 310027, China; 3 Guizhou Anda Aviation Forging Co., Ltd., Anshun 561005, China; zjutsci@163.com

**Keywords:** EIGA, GH4099 alloy powder, selective laser melting, heat treatment, microstructure morphology

## Abstract

GH4099 is a nickel-based, high-temperature, precipitation-strengthened alloy with excellent mechanical properties and corrosion resistance, widely used in aerospace components. The performance of parts produced by additive manufacturing depends significantly on alloy powder quality and heat treatment. In this study, GH4099 alloy powder was prepared using the EIGA method, and its morphology, particle size distribution, and flowability were analyzed. The mechanical properties and microstructure of parts before and after solution-aging treatment were compared. Results showed that the powder had good sphericity and flowability, with a median diameter D50 of 28.88 μm. The formed parts underwent solution treatment at 1140 °C for 2 h followed by aging at 850 °C for 8 h. After heat treatment, the transverse tensile strength increased to 1122.11 MPa (+15.1%) and the yield strength to 866.56 MPa (+22.3%), while the longitudinal tensile strength reached 1116.81 MPa (+29.4%) and the yield strength 831.61 MPa (+35.2%). This improvement is attributed to the precipitation of γ′ phase. Fractographic analysis revealed a mixed fracture mode characterized by ductile dimples and cleavage facets, indicating that the alloy exhibits favorable toughness-related features under mechanical loading. These findings demonstrate the excellent microstructure and mechanical performance of GH4099 alloy in AM applications, providing a basis for its further use in high-performance aerospace components.

## 1. Introduction

GH4099 alloy is a precipitation strengthening high-temperature alloy with nickel as its matrix [1]. The γ′ phase in the alloy acts as the main precipitated phase, providing strengthening effects on the GH4099 alloy and exhibiting excellent corrosion resistance [2]. Furthermore, the solid solution strengthening effects of Co, Mo, and W elements, along with the precipitation strengthening of Al and Ti elements, can significantly enhance the high-temperature performance of the GH4099 alloy, allowing its highest service temperature to reach around 1000 °C with high microstructural stability [3]. Consequently, this alloy is widely used in key components such as aerospace engines, combustion chambers, and blades [4].

In recent years, with the rapid development of additive manufacturing technology, an increasing number of high-temperature alloys have been applied to the manufacturing of complex components in the aerospace field, where their monolithic forming method can fully utilize the various excellent properties inherent to the materials [5]. Selective Laser Melting (SLM) is an additive manufacturing technology that uses metal powder to rapidly melt and solidify under the thermal effect of a laser beam. Compared to traditional processing methods, it has the advantages of high material utilization, short production cycles, and the ability to form complex structures, playing a significant role in the manufacturing of high-strength complex components [6].

Scholars have conducted numerous studies on the application of SLM technology in the manufacturing of complex components. Jia et al. investigated the impact of SLM scanning strategies on the 3D printed parts, and the results indicated that scanning strategies significantly affect the microstructure and mechanical properties of 3D printed components. High energy input and uneven temperature distribution leading to high temperature gradients and thermal stresses can cause significant deformation [7]. Nikolay et al. explored the characteristics of single-component metal powder layers during the SLM process, and the results pointed out that energy and time parameters affect the formation of sintered, semi-sintered/semi-melted, or fully melted blocks, and the dimensions and shape of the components are influenced by the initial powder layer characteristics [8]. Yasa et al. studied secondary processing methods such as selective laser ablation and laser remelting, and the results showed that laser remelting can improve the quality of each layer or the top surface, while selective laser ablation enhances micromachining capabilities [9]. Zhu et al. compared the friction and wear behavior of 316L stainless steel processed by SLM and traditional machining methods, and the results indicated that SLM samples had melt pools, porosity, and fine grains, and exhibited slightly lower friction and wear when in contact with brass compared to traditional samples [10]. The aforementioned studies demonstrate that SLM technology has significant advantages in the manufacturing of complex components, and the process parameters of SLM directly affect the performance and quality of the alloy formed parts.

The performance of alloy formed parts is closely related not only to process parameters but also to many factors, among which the raw material alloy powder is one of the important influencing factors. Currently, the preparation methods for alloy powder are mainly divided into physical-chemical methods and mechanical methods [11], among which the application of atomization methods in mechanical methods is the most widespread, including water atomization [12], gas atomization [13], ultrasonic atomization [14], and centrifugal atomization [12], etc. Gas atomization is widely used in industrial production due to its unique advantages such as high purity and high controllability of powder particle size [13]. Pu et al. prepared CBN/Fe-based spherical magnetic abrasives by gas atomization, which have excellent bonding strength, good sphericity, and processing effects [15]. Yu et al. conducted an in-depth analysis of the characteristics of spherical powder gas atomization preparation technology, pointing out its advantages such as good powder sphericity, strong particle control, high purity, wide applicability, and high production efficiency [16]. The performance of alloy powder has a significant impact on the forming process and the performance of the formed parts, and its performance indicators include surface and internal morphology [17], particle size distribution, and flowability, etc. [18].

Additionally, another crucial factor in the performance of alloy formed parts is the heat treatment process. Appropriate heat treatment process can optimize the internal structure of the alloy, significantly improving its mechanical properties. Currently, numerous studies on the heat treatment processes of alloys have been conducted by researchers. Qu et al. explored the changes in thermal stability, magnetic properties, and mechanical performance of FeCoNiCuTi high-entropy alloy (HEA) after heat treatment at 950 °C for different durations. The study indicated that after heat treatment, the alloy exhibited good thermal stability and significantly enhanced mechanical properties of the alloy [19]. Wu et al. investigated the microstructure and mechanical property changes of CuCrZr alloy produced by SLM during aging heat treatment at different temperatures and durations. The results showed that aging treatment can significantly improve tensile strength, but excessively high temperatures can lead to a decrease in tensile performance [20]. The aforementioned analyses demonstrate that the process differences between SLM manufacturing and traditional processing result in significant differences in the formed microstructures, and the heat treatment process has a significant impact on the microstructure and mechanical properties of the alloy.

Current research on GH4099 alloy mainly focuses on the effects of SLM process parameters and welding interfaces on the alloy’s performance. Lu et al. investigated the microstructure and corrosion resistance of GH4099 alloy produced by SLM under different laser powers and scanning speeds. The results showed that under the conditions of 170 W power and 800 mm/s scanning speed, the samples had the highest relative density, the fewest pores, and the best corrosion resistance [21]. Xiong et al. studied the microstructure and mechanical properties of the joint organization of GH4099 alloy with nickel as its matrix by diffusion welding with a pure nickel interlayer. The results indicated that reducing the interlayer thickness can make the element distribution and hardness more uniform, and the joint organization strength is closer to the original base material and under moderate conditions (1120 °C, 90 min), grain recrystallization eliminated the grain boundaries [22]. It can be seen that current research on GH4099 alloy lacks understanding of the characteristics of SLM raw material alloy powder and is not in-depth enough in studying the impact of heat treatment processes on the organization and performance of SLM formed parts. The characteristics of alloy powder and heat treatment processes have a significant impact on the performance of GH4099 alloy formed parts prepared by SLM and still require further in-depth research.

In this paper, the GH4099 alloy powder was first prepared using the vacuum induction argon gas atomization method; the surface and internal morphology, particle size distribution, and flowability of the alloy powder were deeply studied through a scanning electron microscope (SEM), laser particle size analyzer, hall flow rate meter, and density meter, and other testing instruments. Then, a group of samples were heat treated by a solution aging heat treatment process, and the microstructure of the as-built and solution-aging heat-treated samples were compared. Finally, the mechanical properties and tensile fracture morphology of two different heat-treated samples were compared by tensile test using comprehensive mechanical properties tester and SEM.

## 2. Experimental

### 2.1. Processing and Scan Stratege

**Figure 1:**
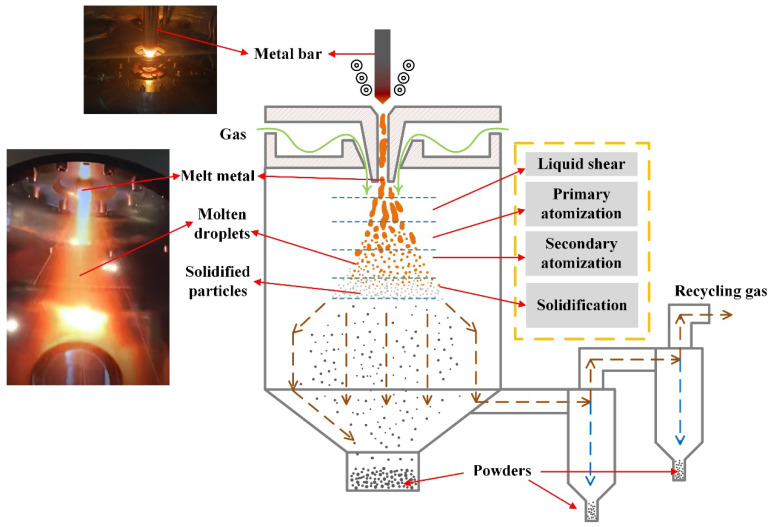
Photos and illustration of EIGA gas atomization processing.

The EIGA Gas Atomization Powder Equipment is composed of the work platform, turret mechanism, melting and insulation chamber, atomization chamber, cyclone powder–gas separation system, vacuum system, water cooling system, gas circuit system, power supply system, electrical control system, detection system, and other auxiliary equipment [23]. From Figure 1, it can be seen that the powder production process of the EIGA Gas Atomization Powder Equipment is as follows: First, the GH4099 alloy bar is placed on the feeder above the high-frequency induction coil, slowly rotating and descending into the induction coil. Under the action of high-frequency induction current, the alloy bar is heated to 1650–1750 °C, and the tip of the bar gradually melts. At this point, the molten droplets, after passing through the guide tube, interact violently with the high-speed inert gas (argon) surrounding the tube. The process of the molten droplets falling can be roughly divided into four stages: the molten droplets stage, the primary atomization stage, the secondary atomization stage, and the cooling and solidification stage. After cooling and solidification, part of the formed alloy powder directly falls into the main powder tank, while the finer alloy powder is carried into the secondary tank by the action of the secondary cyclone classification system, which also recovers the inert gas.

**Figure 2:**
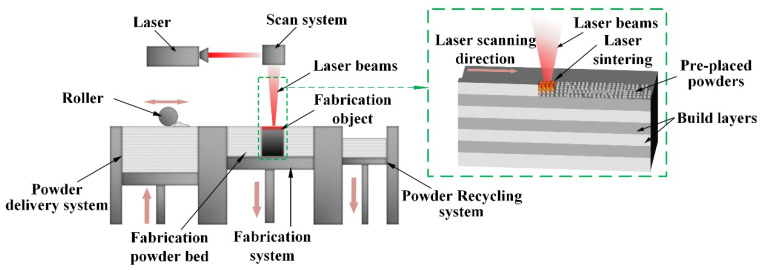
Illustration of SLM processing.

The SLM additive manufacturing method was employed in this paper. The SLM method utilizes the laser energy source, following the path planned in the 3D CAD sliced model to scan the metal powder bed layer by layer. The scanned metal powder achieves metallurgical bonding through melting and solidification, ultimately obtaining the metal parts designed in the model. The schematic diagram of the technical principle is shown in Figure 2.

### 2.2. GH4099 Powder

GH4099 alloy powder is prepared using the EIGA method, the appearance of the alloy powder is generally regular and spherical and the particle size distribution is uniform. To reduce the moisture in the powder and increase its flowability, the GH4099 alloy powder is dried in a vacuum chamber for 8 h at a temperature of 180 °C. After the drying is completed, it is cooled to room temperature inside the vacuum chamber before being removed. The elemental distribution of the GH4099 alloy powder is shown in Table 1.

### 2.3. Characterization Methods of Powder and 3D Printed Products

**Figure 3:**
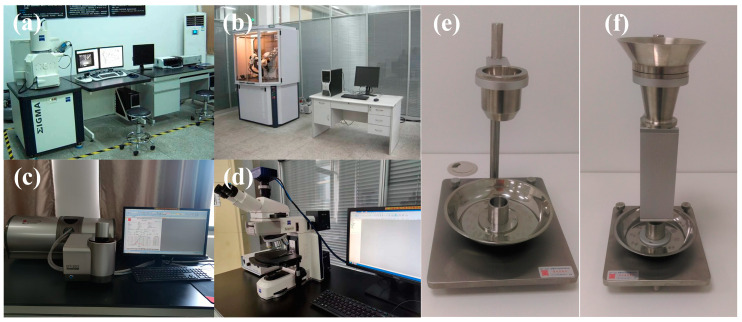
Performance characterization test instruments: (**a**) ZEISS Sigma 300 Scanning electron microscope (Jena, Germany), (**b**) Bruker D8 Advance X-ray diffractometer (Karlsruhe, Germany), (**c**) Bettersize BT-802 Laser particle size analyzer, (**d**) ZEISS Scope A1, (**e**) Bettersize BT-200 Hall flowmeter (Dandong, China), (**f**) Bettersize BT-101 Density tester.

GH4099 alloy powder was prepared using the EIGA Gas Atomization method. Figure 3 shows the performance characterization test instruments. The ZEISS Sigma 300 scanning electron microscope (Jena, Germany) was used to scan the evenly spread GH4099 alloy powder particle samples. A total of two sets of scanning electron microscope images were taken. The first set was scanned with an Extra High Tension (EHT) of 15.00 kV and a magnification (Mag) of 200×, obtaining the overall morphology distribution of the powder. The second set was scanned with an Extra high tension (EHT) of 15 kV, a working distance (WD) of 8.1 mm, and a magnification (Mag) of 500×, yielding a clearer surface morphology of the alloy powder. The ZEISS METROTOM 800 was used for tomography scanning of the powder samples, obtaining 2D tomographic images, thereby displaying the hollow powder images of the alloy powder. The BT-802 model laser particle size analyzer from Dandong Bettersize Company (Dandong, China) was used to statistically analyze the particle size distribution of a portion of the samples, obtaining preliminary distribution patterns of the powder particle sizes. The BT-200, BT-101, and BT-310 model hall flow rate meters from Dandong Bettersize Company were used to measure the hall flow rate, bulk density, and tapped density of the powder, yielding preliminary results. The Bruker D8 Advance model X-ray diffraction (XRD) analyzer produced by Bruker Corporation (Karlsruhe, Germany) was used for the XRD analysis of some samples of the alloy powder.

3D Printing employs SLM technology to prepare samples, with detailed process parameters as shown in Table 2. To ensure the stability of the forming process and the reliability of the validation data, all samples are prepared using the same 3D printing process parameters. The prepared GH4099 alloy samples are divided into two groups, as shown in Table 3. The first group is left without any treatment (as-built state), while the second group undergoes a solution treatment at 1140 °C for 2 h followed by an aging treatment at 850 °C for 8 h with furnace cooling (HT state). After that, both groups of samples are ground and polished, and SEM is used to observe their X–Y section metallographic microstructures.

**Figure 4:**
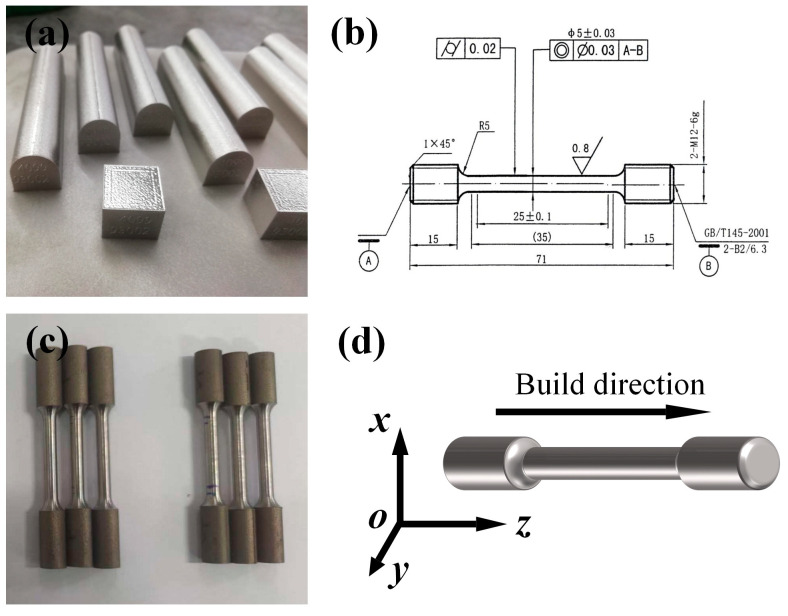
(**a**) Printed finished product images, (**b**) tensile test bar shape and dimensions [25], (**c**) as-built and heat-treated tensile test bars, (**d**) sampling and tensile direction of the tensile test bars.

The mechanical property testing of the samples is conducted using M12 × 71 mm standard specimens, as shown in Figure 4b, in accordance with the Chinese standard GB/T 228.1-2021 “Metallic materials—Tensile testing—Part 1: Method of test at room temperature” [24]. Both groups of specimens undergo the heat treatment methods described previously for tensile fracture testing at room temperature. The mechanical property testing of the samples is carried out in two directions, transverse and longitudinal, as shown in Figure 4d, with the O–Z direction being the longitudinal direction and the X–Y direction being the transverse direction. The tensile testing analysis mainly includes tensile strength, yield strength, and elongation after fracture. Each group of specimens has the transverse and longitudinal tests repeated three times to eliminate randomness.

## 3. Results and Discussion

### 3.1. Properties Characterization of GH4099 Alloy Powder

#### 3.1.1. Morphology Analysis and Particle Diameter Distribution of GH4099 Alloy Powder

**Figure 5:**
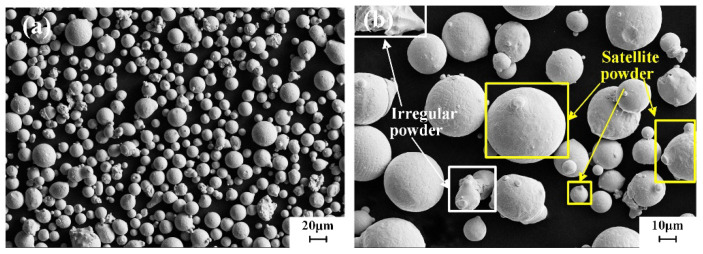
GH4099 powder SEM image: (**a**) morphology of the powders (**b**) defective powders.

**Figure 6:**
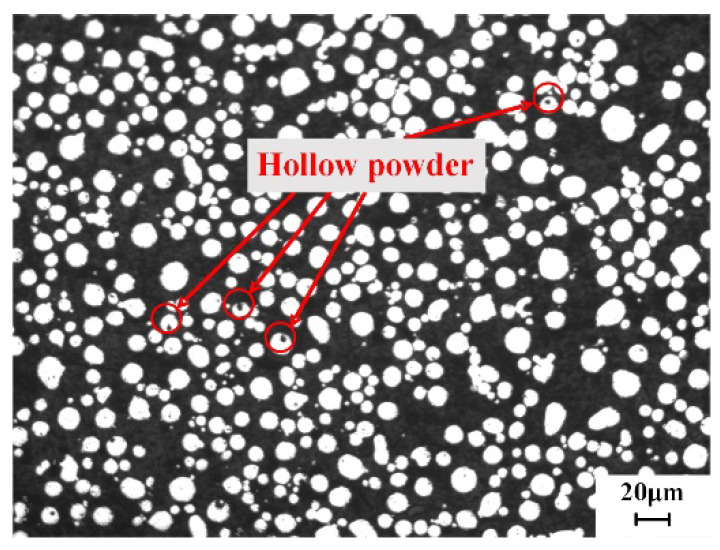
GH4099 powder light microscope image.

The SEM morphology of the GH4099 alloy powder is shown in Figure 5. The overall particle size distribution is between 20 and 40 μm, exhibiting good sphericity and surface smoothness, with a small amount of irregular powder and satellite powder present. Additionally, there are instances of powder agglomeration. The appearance of satellite powder is primarily due to the slower solidification rate of larger molten droplets compared to smaller ones. During the flight process, smaller molten droplets solidify more quickly than larger ones and collide with the larger droplets, leading to the formation of satellite powder on the surfaces of the larger droplets. The presence of satellite powder and irregular particles will significantly affect the performance of the alloy powder. Figure 6 displays the CT cross-sectional image of the GH4099 alloy powder. It can be observed that there are a few hollow particles within the alloy powder. The formation of these hollow particles is attributed to the strong two-phase interaction between high-pressure gas and molten metal. After the molten liquid breaks into droplets, they solidify rapidly, preventing gas from escaping, which results in the gas being trapped inside the alloy powder, forming hollow particles. During the subsequent 3D printing process, hollow particles will lead to a reduction in the density of the printed parts and the formation of internal pores, severely affecting their mechanical properties.

Table 4 presents the comprehensive properties of the GH4099 alloy powder, with measurements taken using a Hall flowmeter. The Hall flow rate is an important indicator of powder flowability, representing the time it takes for 50 g of powder to completely pass through the flowmeter funnel; a smaller Hall flow rate value indicates better powder flowability [26]. Several factors influence powder flowability, including particle size, surface morphology, and surface roughness [27]. Generally, larger particle sizes lead to better flowability; however, smaller particles, having a larger specific surface area and greater contact area, exhibit more pronounced interaction forces, hindering flow [28]. Nevertheless, larger particle sizes are not always preferable. As previously mentioned, factors such as surface morphology and roughness also significantly affect powder flowability. Typically, powders with excessively large particle sizes (>100 μm) are accompanied by poor surface morphology and irregular shapes, negatively impacting flowability. During the SLM forming process, larger particle sizes are more prone to incomplete melting under laser energy, resulting in internal voids and reduced density, thereby degrading the performance of the printed product. As shown in Table 5, the Hall flow rate of the GH4099 alloy powder is 14.9 ± 0.02 s/50 g, indicating good flowability and suggesting that the powder’s surface morphology and roughness are within a favorable range. The tapped density and bulk density also reflect the powder’s flowability, measured at 5.32 ± 0.042 g/cm^3^ and 4.68 ± 0.023 g/cm^3^, respectively, with a ratio close to 1, further indicating good flowability.

**Figure 7:**
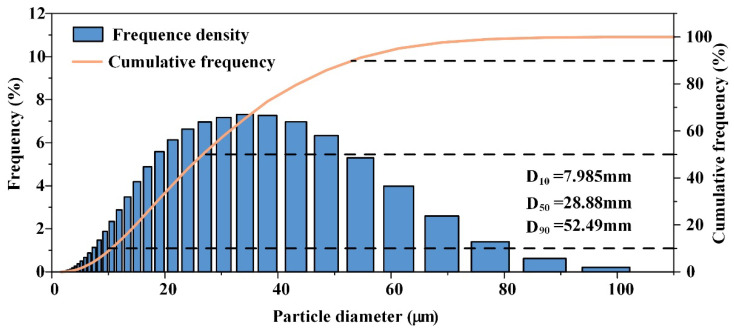
Particle Size Distribution of GH4099 Alloy Powder.

The particle size distribution of the GH4099 alloy powder is shown in Figure 7. The histogram indicates a unimodal distribution that closely approximates a normal distribution. The cumulative particle size distribution curve reveals that over 80% of the powder has a particle size of ≤40 μm. The average particle size, represented by the D_50_ value corresponding to a cumulative mass distribution of 50%, is calculated to be 28.88 μm. Similarly, the D_10_ and D_90_ values are determined to be 7.985 μm and 52.49 μm.

#### 3.1.2. EDS Spectrum and XRD Pattern Analysis of GH4099 Alloy Powder

**Figure 8:**
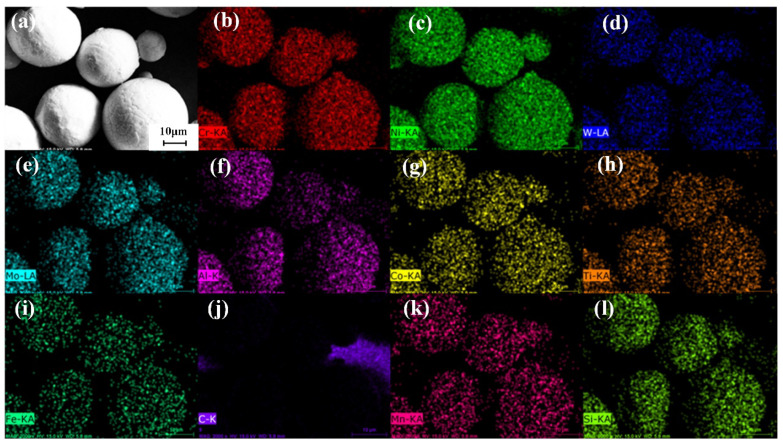
EDS energy spectrum mapping of GH4099 alloy powder. (**a**) SEM image of the morphology of the powder, (**b**) Cr (Chromium), (**c**) Ni (Nickel), (**d**) W (Tungsten), (**e**) Mo (Molybdenum), (**f**) Al (Aluminum), (**g**) Co (Cobalt), (**h**) Ti (Titanium), (**i**) Fe (Iron), (**j**) C (Carbon), (**k**) Mn (Manganese), (**l**) Si (Silicon).

As shown in Figure 8, the EDS spectrum of GH4099 alloy powder indicates that the powder microstructure primarily consists of dendrites and cellular crystals. When the powder particles are sufficiently small, amorphous structures can also be observed. Larger particles exhibit satellite particles and burrs on their surfaces, while smaller particles have smoother surface structures. The differences in surface morphology among powders of varying particle sizes are mainly attributed to different cooling rates: larger particles have longer spheroidization and cooling times, resulting in a relatively low solidification rate. Their larger volume leads to more significant shrinkage, leaving noticeable irregular shrinkage marks on the surface after the atomized molten droplets solidify. In contrast, smaller particles spheroidize and cool more rapidly due to their shorter time frames, resulting in quick solidification under the influence of surface tension, which forms particles with an amorphous surface structure. During their descent, collisions occur between solidified small particles and larger particles that have not completely solidified, leading to the formation of satellite powder and burrs. The atomization process of the alloy melt occurs rapidly, without introducing other harmful impurities.

**Figure 9:**
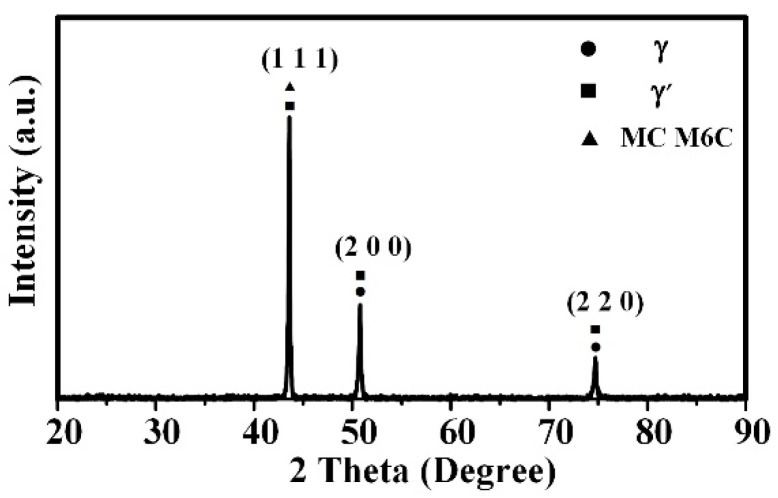
XRD pattern of GH4099 alloy printed products.

As illustrated in Figure 9, the GH4099 high-temperature alloy powder exhibits a face-centered cubic (FCC) structure, with the matrix being austenite. Elements such as Ni, Cr (a = 0.3589 nm, space group Pm-3m), Co, W, Al, and Ti contribute to the overall strengthening of the alloy. The γ′ phase serves as the primary strengthening phase, co-precipitating coherently with the γ phase (NiCoCr)_3_(AlTiCrW). The diffraction peaks of the γ′ phase overlap with those of the γ phase. In the GH4099 powder, there is a preferred orientation along the (1 1 1) crystal plane, and the powder texture primarily grows along the (1 1 1) plane. The strengthening elements Co, W, Al, and Ti are evenly distributed within the Cr, Fe, and Ni matrix, resulting in a uniform composition without segregation (Figure 8b–l).

### 3.2. Mechanical Properties of Printed Products

#### 3.2.1. Microstructure Metallographic Analysis

**Figure 10:**
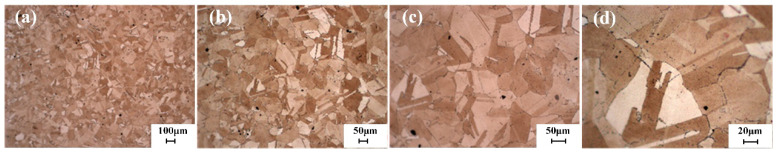
Microstructure of the As built state. (**a**) image at a MAG of 100 μm, (**b**) image at a MAG of 50 μm, (**c**) image at a MAG of a different scale length 50 μm, (**d**) image at a MAG of 20 μm.

Before solution treatment, the microstructure of the GH4099 alloy in its as built state is shown in Figure 10. It can be observed that the irregular blocky structure corresponds to the γ phase, or austenite, which features large grain sizes. Due to thermal processing, some grains exhibit a certain degree of deformation and have not undergone complete recrystallization. Additionally, there are significant large particle precipitates present within the grains, characteristic of the material prior to solution heat treatment.

**Figure 11:**
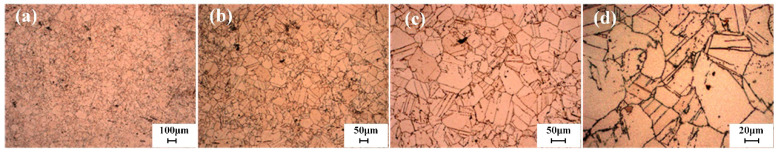
Microstructure of the HT state. (**a**) image at a MAG of 100 μm, (**b**) image at a MAG of 50 μm, (**c**) image at a MAG of a different scale length 50 μm, (**d**) image at a MAG of 20 μm.

The metallographic structure after solution treatment at 1140 °C for 2 h followed by aging at 850 °C for 8 h is shown in Figure 11. After solution treatment, the matrix γ phase transforms from elongated columnar grains to equiaxed grains, with most of the larger precipitates having dissolved. Within the grains, a fine dispersion of the second phase, γ′ phase, precipitates, which is an important strengthening phase for the alloy. The large precipitate particles in the GH4099 alloy completely dissolve into the matrix at 1140 °C, and during aging, the γ′ phase re-precipitates. When the solution temperature exceeds 1100 °C, the dissolution of the γ′ phase weakens the restriction on grain growth, leading to the disappearance of dendritic morphology within the grains. Concurrently, numerous twin boundaries appear within the grains. The high solution temperature of 1140 °C accelerates the migration rate of grain boundaries, while a large number of alloying elements dissolve into the matrix, reducing the resistance to grain boundary migration during grain growth and significantly accelerating the rate of grain coarsening.

Furthermore, the higher the solution treatment temperature, the better the solubility of the alloying elements, which enhances the effectiveness of the subsequent aging treatment [29]. After aging at 850 °C for 8 h, a large number of secondary γ′ precipitates form within the grains. The GH4099 alloy has a face-centered cubic (FCC) crystal structure, and the formation of twins occurs during the high-temperature solution process, representing typical annealing twins. Moreover, high-temperature solution treatment promotes homogenization, allowing elements like Al and Ti to fully dissolve in the γ matrix. The aging temperature of 850 °C is relatively low, insufficient to cause recrystallized grain growth. Prolonged aging leads to the precipitation of elements such as Al, Ti, Cr, and W in the form of γ′, resulting in a more uniform microstructure after the solution-aging treatment of GH4099.

#### 3.2.2. Tensile Mechanical Properties

**Figure 12:**
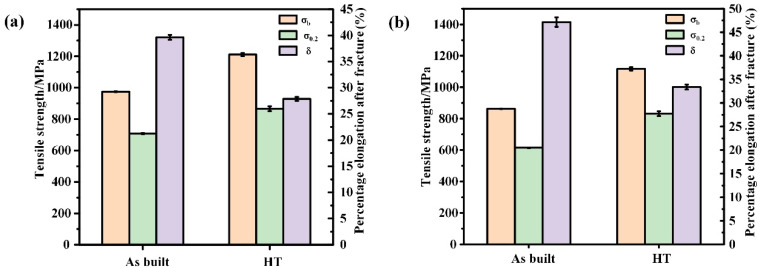
Microstructure of the printed state and HT state: (**a**) transverse tensile properties at room temperature, (**b**) longitudinal tensile properties at room temperature.

The comparison of transverse and longitudinal tensile properties between the printed state and heat-treated state at room temperature is shown in Figure 12. The transverse tensile strength of the printed samples can exceed 974.74 MPa, with a yield strength of 708.53 MPa and a post-fracture elongation of over 39.64%. In contrast, the longitudinal tensile strength reaches above 862.97 MPa, with a yield strength of 614.74 MPa and a post-fracture elongation of over 47.17%. Similar to other alloys, the mechanical properties of the GH4099 samples produced by SLM exhibit anisotropy, with transverse strength outperforming longitudinal strength and lower plasticity compared to the longitudinal direction.

**Figure 13:**
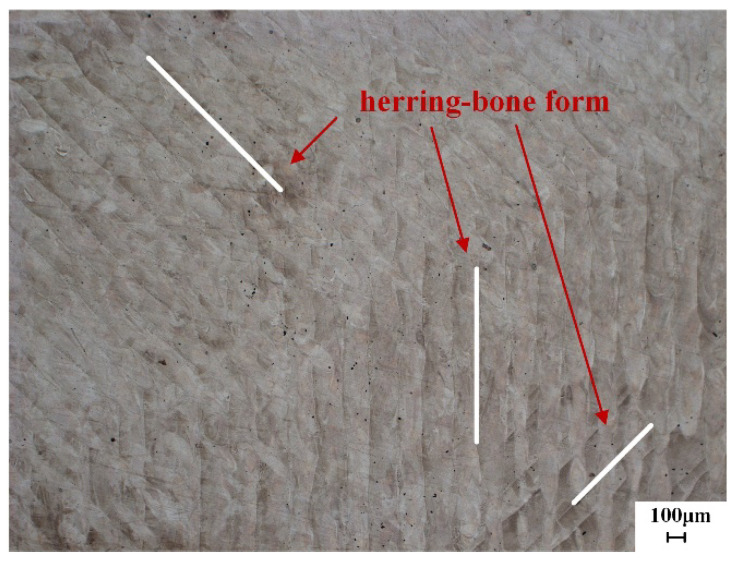
Morphology of the welding bead in the transverse X–Y direction.

In the transverse X–Y direction, the overlapping melt pools create a dense microstructure without significant unmelted defects. The micro melt pools stack in a fish-scale pattern along the laser deposition direction (Figure 13). During the laser cycling process, local overlap occurs in the melt pools, leading to significant changes in dendritic orientation and forming a distinct banded structure.

In the longitudinal O–Z plane, both tensile strength and yield strength are lower than those of the transverse samples. The banded structure formed during deposition can easily lead to element segregation and other inclusions, which may serve as crack initiation sources during deformation. This anisotropy is attributed to the different temperature gradients in various directions during the SLM process, resulting in distinct microstructures. In general applications, such anisotropy should be avoided.

After solution treatment at 1140 °C for 2 h and aging at 850 °C for 8 h, the transverse tensile strength reaches 1122.11 MPa, with the yield strength increasing to 866.56 MPa. The longitudinal tensile strength achieves 1116.81 MPa, with a yield strength of 831.61 MPa. In the GH4099 alloy, the γ′ phase is the primary contributor to the alloy’s strength, while the secondary γ′ phase impedes dislocation motion, thereby enhancing yield strength. Furthermore, the heat treatment process results in a more uniform microstructure and reduces anisotropy in tensile properties. However, it is also accompanied by a decline in elongation, indicating a trade-off between improved strength and reduced ductility. This behavior is commonly observed in strengthened superalloys where hardening mechanisms limit plastic deformation.

Thus, heat treatment significantly affects the quantity and size of the γ′ phase, dislocations, and twins in the microstructure, which are crucial factors influencing the overall performance of the manufactured parts. The solution treatment at 1140 °C for 2 h followed by aging at 850 °C for 8 h can markedly improve the strength of SLM high-temperature alloy components, though some loss in plasticity may occur.

#### 3.2.3. Tensile Fracture Microstructure Morphology

**Figure 14:**
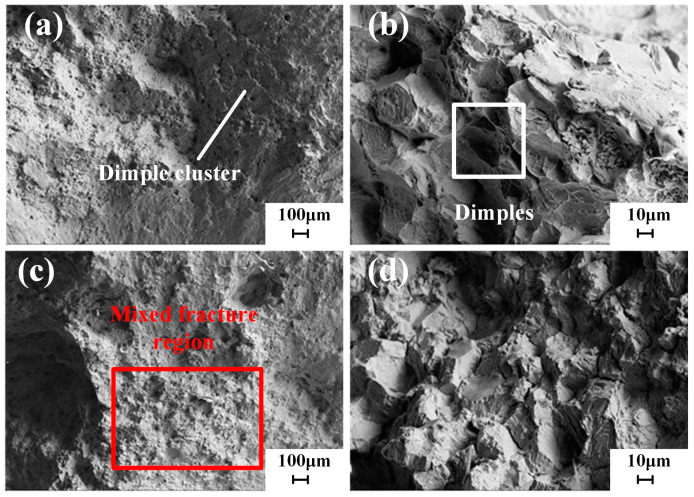
Microstructure of the tensile fracture in the as built state (SEM): (**a**,**b**) microstructure of the fracture in the X–Y direction, (**c**,**d**) microstructure of the fracture in the O–Z direction.

**Figure 15:**
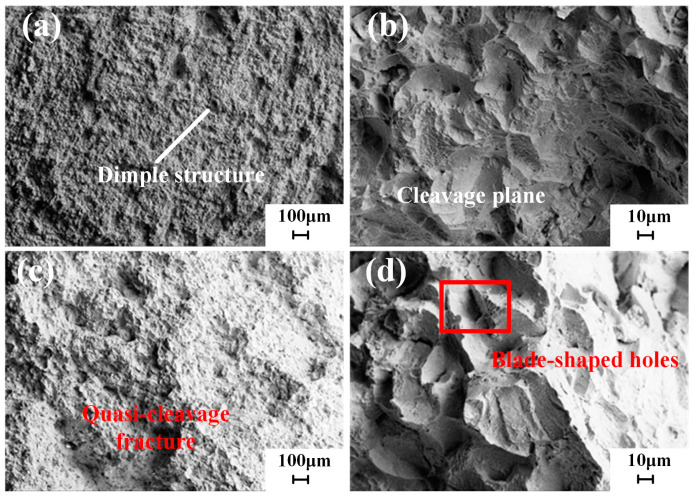
Microstructure of the tensile fracture in the HT state (SEM): (**a**,**b**) microstructure of the fracture in the X–Y direction, (**c**,**d**) microstructure of the fracture in the O–Z direction.

The observation of the tensile fracture morphology of SLM-formed samples is shown in Figure 14 and Figure 15. The microstructure of the tensile fracture features dimples and cleavage planes, indicating a mixed ductile–brittle quasi-cleavage fracture characteristic. The tensile fracture morphology of the as-built samples displays numerous dimples of varying sizes and depths shown in Figure 14, exhibiting characteristics of ductile fracture. The formation of dimples primarily arises from the stress concentration caused by voids and other inclusions within the printed components during the tensile process. When the applied stress reaches the material’s yield limit, plastic deformation occurs, creating crack initiation sites at the defects, which then propagate and form a dimpled fracture morphology. This deformation initially leads to the formation of micropores in regions experiencing significant plastic deformation. Dimples are the manifestation of micropore nucleation, growth, and coalescence at the fracture surface, representing a significant feature of ductile fracture.

After solution aging heat treatment, the dimples at the fracture surface become smaller and shallower, accompanied by the appearance of numerous cleavage planes, as shown in Figure 15. A few blade-shaped holes can be observed. These holes result from two primary factors: some are generated during the tensile testing process, while others arise during the SLM process. During the remelting process, some larger particles may not completely melt, resulting in the formation of pores within the component. Since SLM is a layer-by-layer deposition process, the surface of the previous layer is rough after melting and solidification, preventing the subsequent layer of powder from adequately filling the gaps, ultimately leading to the formation of voids at the tensile fracture. This effect causes a notable anisotropy in the mechanical properties of the samples in both the transverse and longitudinal directions. The tensile strength of the specimens has significantly improved. During high-temperature solution treatment, the strengthening phases gradually dissolve, and during the aging process, the grains refine, resulting in a more uniform microstructure. The strengthening elements in the γ matrix significantly enhance the alloy’s tensile strength, while the growth of carbide precipitates at the grain boundaries leads to a reduction in boundary strength, creating weakened regions. Cracks preferentially initiate between the carbides and the matrix and propagate along the grain boundaries, forming crack sources and causing a decrease in alloy ductility. Additionally, heat treatment effectively eliminates some of the melt pool boundaries, resulting in a more consistent ductility in both the transverse and longitudinal directions.

### 3.3. Discussion

The GH4099 alloy powder prepared in this study exhibits high sphericity, smooth surface morphology, and a concentrated, relatively small particle size distribution (D_50_ = 28.88 μm), which contributes to enhanced powder spreading stability during SLM. Compared to the Inconel 625 powder (D_50_ = 30 μm) reported by Ur Rehman et al. [30] and the Inconel 718 powder (D_50_ = 32 μm) studied by Nguyen et al. [31], the powder in this study has a slightly smaller particle size and superior morphology. The Hall flow rate was measured at 14.9 s/50 g, which is slightly better than the flow rate of 16–17 s/50 g reported for Inconel 718 powder used in SLM by Park et al. [32]. EDS analysis shows a uniform distribution of alloying elements, while XRD results reveal that the powder predominantly consists of face-centered cubic *γ* phase, consistent with the findings of Zhou et al. [33] and Deng et al. [34]. Additionally, a small amount of satellite and hollow particles were observed in the powder, which are common defects in the gas atomization process and may negatively impact the subsequent additive manufacturing quality. This observation aligns with the results of Zhou et al. [33] and Deng et al. [34], indicating that further optimization of the atomization parameters is needed to reduce the defect rate.

The GH4099 alloy exhibits anisotropic mechanical properties in its as-printed state, with transverse strength surpassing longitudinal strength, which is consistent with findings in alloys such as Inconel 718 and Inconel 625 [35]. Heat treatment enhances the strength of GH4099 alloy and reduces anisotropy, as observed in other superalloys [36]. However, it also leads to a slight decrease in ductility, reflecting the trade-off between strength and plasticity commonly seen in strengthened alloys.

Based on the above discussion, the key findings regarding powder quality, mechanical anisotropy, and heat treatment effects are summarized as follows. The GH4099 alloy powder exhibits excellent quality for SLM, and the as-printed samples show significant mechanical anisotropy. Heat treatment effectively enhances strength and reduces anisotropy, consistent with trends observed in other superalloys.

## 4. Conclusions

This paper characterizes the key performance indicators of GH4099 alloy powder prepared by vacuum induction argon atomization, including surface morphology, internal structure, fluidity, and other properties. Various heat treatments were applied to SLM-manufactured samples, and their microstructure, mechanical properties, and fracture morphology were analyzed. The main conclusions are summarized as follows:

(1) The particle size distribution of GH4099 alloy powder is primarily between 20 and 40 μm, with a D50 of 28.88 μm and a Hall flow rate of 14.9 ± 0.02 s/50 g, indicating good sphericity, surface smoothness, and flowability. However, a small amount of satellite and hollow powders is present.

(2) The microstructure of GH4099 powder mainly consists of dendrites and cellular structures, exhibiting a face-centered cubic (FCC) crystal structure. The matrix is austenitic, with a preferred orientation of (1 1 1). Reinforcing elements such as Co, W, Al, and Ti are uniformly distributed within the Cr, Fe, and Ni matrix, showing no segregation.

(3) After solution treatment and aging, the matrix γ-phase of GH4099 alloy transforms from columnar grains to equiaxed grains, promoting the re-precipitation of fine γ’ phases and enhancing the alloy’s performance. Higher solution treatment temperatures and aging effectively lead to a uniform microstructure, significantly improving the mechanical properties of the alloy.

(4)The mechanical performance of the untreated GH4099 high-temperature alloy at room temperature is suboptimal. However, after undergoing a heat treatment at 1140 °C for 2 h followed by aging at 850 °C for 8 h, the mechanical properties of GH4099 alloy improve significantly, with transverse tensile strength and yield strength increasing by 15.1% and 22.3%, respectively, and longitudinal tensile strength and yield strength increasing by 29.4% and 35.2%.

(5) In the printed state, the GH4099 alloy exhibits mixed ductile–brittle fracture characteristics due to the presence of voids and stress concentrations. However, the solution aging heat treatment significantly increases the size of dimples and tensile strength, leading to grain refinement and microstructural uniformity, which greatly enhances the mechanical performance of the alloy.

## Figures and Tables

**Table 1:** Chemical composition of GH4099 alloy powder.

Element	Cr	W	Co	Mo	Al	Ti	Fe	Ni
Proportion	17.94	6.02	5.8	3.93	2.24	1.19	0.2	Bal.

**Table 2:** SLM scan parameters.

Parameters	Unit	Value
Layer thickness	μm	20
Overlap	%	3
Scan velocity	mm/s	1095
Laser power	W	190
Initial temperature	°C	140
Scan strategy	-	Short line
Grain boundary energy	J/m^2^	0.25150

**Table 3:** Heat treatment methods for the printed products.

Condition	Heat Treatment
As built	-
HT	1140 °C/2 h/solution + 850 °C/8 h aging treatment

**Table 4:** Heat treatment methods for the printed finished products.

Condition	Heat Treatment
As built	-
HT	1140 °C/2 h/solution + 850 °C/8 h aging treatment

**Table 5:** Comprehensive properties of GH4099 powder.

Hall Flow Rate/50 g	Tapped Density g/cm^3^	Bulk Density g/cm^3^
14.9 ± 0.02	5.32 ± 0.042	4.68 ± 0.023

## Data Availability

The original contributions presented in the study are included in the article, further inquiries can be directed to the corresponding authors.
